# Long-term *in vivo* dissolution of thermo- and pH-responsive, ^19^F magnetic resonance-traceable and injectable polymer implants†

**DOI:** 10.1039/d4na00212a

**Published:** 2024-04-08

**Authors:** Natalia Jirát-Ziółkowska, Martin Vít, Ondřej Groborz, Kristýna Kolouchová, David Červený, Ondřej Sedláček, Daniel Jirák

**Affiliations:** a Radiodiagnostic and Interventional Radiology Department, Institute for Clinical and Experimental Medicine Videnska 1958/9 140 21 Prague Czech Republic daniel.jirak@ikem.cz +420-736467349; b Institute of Biophysics and Informatics, First Faculty of Medicine, Charles University Katerinska 1660/32 Prague 121 08 Czech Republic; c Institute of Macromolecular Chemistry, Czech Academy of Sciences Heyrovsky square 2 162 06 Prague Czech Republic; d Faculty of Health Studies, Technical University of Liberec Studentska 1402/2 Liberec 461 17 Czech Republic; e Department of Physical and Macromolecular Chemistry, Faculty of Science, Charles University Hlavova 8 Prague 128 00 Czech Republic

## Abstract

^19^F magnetic resonance (^19^F MR) tracers stand out for their wide range of applications in experimental and clinical medicine, as they can be precisely located in living tissues with negligible fluorine background. This contribution demonstrates the long-term dissolution of multiresponsive fluorinated implants designed for prolonged release. Implants were detected for 14 (intramuscular injection) and 20 (subcutaneous injection) months by ^19^F MR at 4.7 T, showing favorable MR relaxation times, biochemical stability, biological compatibility and slow, long-term dissolution. Thus, polymeric implants may become a platform for long-term local theranostics.

## Abstract 2

Thermo- and pH-responsive polymers' dissolution *in vivo* can be broadly fine-tuned. Polymeric implant can be detected *in vivo* using “hot-spot” ^19^F MR measurement. Long-term *in vivo* dissolution of polymeric implant shows no side effects.

## Graphical abstract

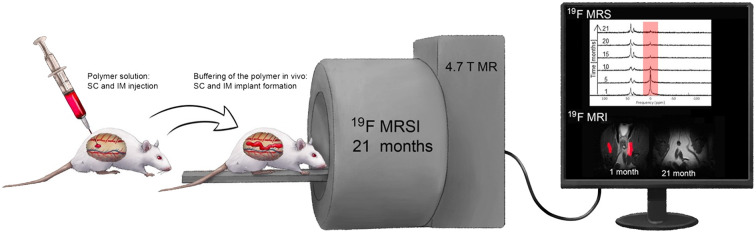

## 1. Introduction

Magnetic resonance spectroscopy and imaging (MRS/MRI) are irreplicable, non-invasive methods. These methods do not use ionizing radiation and are routinely applied in clinical practice. ^1^H MRI enables clinicians and researchers to diagnose diseases and anatomical anomalies. However, some pathological alterations may be difficult to observe in native ^1^H MRI scans due to low contrast between those changes and healthy tissues. To overcome this problem, contrast agents that accumulate in pathological tissues may be administered, thereby broadening the scope of MRI applications in clinical practice. Nevertheless, most clinically approved MR contrast agents contain gadolinium,^1^ which may accumulate in organisms and cause considerable side effects. Therefore, non-gadolinium-based contrast agents or tracers are highly desirable for future MR applications.

Another approach to broadening MR applications is the development of new contrast agents,^2^ based on other nuclides, *i.e.*, heteronuclear (X-nuclei) MR.^3,4^ The most promising X-nuclei is ^19^F for its high sensitivity (83% of that for ^1^H^5^). Additionally, as ^19^F is the only naturally occurring isotope of fluorine, no isotope enrichment is required. Another advantage is that ^19^F tracers can be highly biocompatible and non-toxic^6–13^ and have a broad range of chemical shifts (−300 to 400 ppm), so multiple tracers can be simultaneously tracked with a higher sensitivity than other X-nuclei. Moreover, dual ^1^H/^19^F MR can be acquired using common scanners and radiofrequency coils with only minor hardware adjustments.^14^ Lastly, the physiological concentration of fluorine is negligible^6,7^ and therefore ^19^F MR is a very specific method. For these reasons, any administered fluorinated xenobiotic can be traced and overlaid with a ^1^H MRI signal for anatomical placement, and its signal is proportional to the number of ^19^F nuclei, thus allowing the absolute quantification of fluorine.^8^

The applicability of ^19^F MR relies heavily on suitable ^19^F MR tracers. Although many experimental fluorine tracers have been developed already,^6–13,15–17^ only a few are clinically applicable. Recently, we developed polymer tracers based on thermoresponsive poly[*N*-(2,2-difluoroethyl)acrylamide] (PDFEA).^16^ PDFEA is biocompatible and hydrophilic and has a high content (28 wt%) of magnetically equivalent fluorine atoms with highly suitable ^19^F MR relaxation times. As such, PDFEA copolymers are ideal ^19^F MRI tracers.^7,18,19^ Moreover, the type and content of co-monomers may be used to broadly fine-tune the properties of the polymers and to induce additional stimuli-responsiveness.^20^ Based on the above, PDFEA may become a polymer platform for developing smart ^19^F MRI tracers.

In this study, we report the results from our long-term MR monitoring and analysis of a multi-stimuli-responsive PDFEA copolymer-based implant, highlighting its potential as a smart ^19^F MRI tracer for parenteral applications. As described in our previous study,^7^ this polymer was designed to form an insoluble hydrogel at pH = 7.4 and 37 °C (physiological conditions) but remain soluble at lower pH or temperatures. Such a polymer can be dissolved in a slightly acidic environment (pH = 5.0) at room temperature and administered to the body, where its solution is neutralized and heated above the critical temperature, forming an insoluble implant *in situ*. Here, we show that this polymeric implant remains at the administration site for months (depending on its composition) and can function locally.^21^ Accordingly, drugs may be incorporated into the implant structure or co-administrated with the carrier,^7^ which is released over time, thereby increasing the efficacy of the therapy and limiting its systemic side effects.^22–31^ Given the high fluorine content of this polymer, its dissolution can be monitored by ^19^F MRI, with numerous clinical and biomedical applications. Our findings confirm the potential of this polymer as an advanced theranostic tracer for long-term ^19^F MR diagnostics.

## 2. Materials and methods

### 2.1 Polymer synthesis and characterization

The tracer was synthesized as in our previous study^7^*via* reversible addition–fragmentation chain transfer (RAFT) copolymerization of the corresponding monomers. The monomers DFEAM and *N*-[3-(1*H*-imidazole-1-yl)propyl]acrylamide (ImPAM) were synthesized according to the ref. 16. All other chemicals, including *N*-(2-hydroxyethyl)acrylamide (HEAM), were purchased from Sigma-Aldrich. The number-average molecular weight (*M*_n_), weight-average molecular weight (*M*_w_), and polymer dispersity (*Đ* = *M*_w_/*M*_n_) were analyzed by SEC on an HPLC Ultimate 3000 system (Dionex, Sunnyvale, USA) equipped with an SEC column (TSKgel SuperAW3000 150 × 6 mm, 4 μm). Three detectors, UV/Vis, refractive index (RI) Optilab®-rEX and multiangle light scattering (MALS) DAWN EOS (Wyatt Technology Co., USA), were used with a methanol and sodium acetate buffer (0.3 M, pH 6.5) mixture (80 : 20 v/v, flow rate of 0.6 mL min^−1^) as the mobile phase.

Copolymer composition was determined by ^1^H NMR on a Bruker Avance III 400 MHz NMR spectrometer (Bruker, Rheinstetten, Germany). The thermoresponsive behavior of the copolymer was studied by turbidimetry in buffered aqueous solutions, where the cloud point temperature (*T*_CP_) was indicated by a decrease in sample transmittance below 90%. The polymer (*c*_pol_ = 5.0 mg mL^−1^) was dissolved in 150 mM phosphate buffer-saline (pH = 7.0) or 150 mM sodium acetate buffer (pH = 5.0). Transmittance was measured at 600 nm on a Thermo Scientific Evolution 220 UV/VIS spectrophotometer equipped with a Thermo Scientific single-cell Peltier element (Thermo-Fisher, Waltham, USA). At temperature increments of 0.1 °C, the samples were stirred at 700 rpm. The effects of slight changes in pH and temperature on the MR signal were described in Sedlacek *et al.*, 2018 and our setup was based on those findings.^16^

For *in vivo* use, all copolymers had a narrow molecular mass distribution (dispersity *Đ* ≤ 1.20), and their molar mass was approximately 40 kDa. Polymers with lower critical solution temperature (LCST) properties are eliminated through the urine and bile when their LCST is well below body temperature due to the balance between their dissolved and non-dissolved phases. The polymers were designed to dissolve at pH 5.0, at which the *T*_CP_ value of the copolymer must be well above body temperature to prevent obstruction of the needle by polymer aggregation during administration. In turn, *T*_CP_ must be below the body temperature at pH 7.4 to enable the rapid formation of the implant.

### 2.2 ^1^H/^19^F MR coil homogeneity

In this study, we used a custom circular ^1^H/^19^F radiofrequency (RF) surface single-loop coil, with a diameter of 4 cm, optimized for small laboratory animals experiments on a 4.7 T MR scanner (Bruker BioSpec 47/20, Ettlingen, Germany). The coil was designed to enable on-machine tuning and matching at ^1^H and ^19^F Larmor frequencies. To examine ^1^H/^19^F MR RF coil homogeneity performance, we conducted a homogeneity test on a water-filled phantom. In all measurements, we used a gradient-echo ^1^H MRI sequence with low flip angles (fast low angle shot FLASH; flip angle FA = 10°; repetition time/echo time TR/TE = 171.8/3.7 ms; number of averaging NA = 1; field of view FOV = 80 × 80 mm; digital matrix 256 × 128; number of slices 20 in transversal, sagittal and coronal plane; time of acquisition TA = 22 s). Secondly, coil homogeneity was evaluated using Matlab software (Matlab R2007b; The MathWorks, Inc., USA) script by adding artificial colors reflecting the signal attenuation in decibels (dB) with maximum signal intensity referenced as 0 dB. The FOV used in the phantom measurement in the coronal plane exceeded that used in ^19^F *in vivo* experiments (FOV = 80 × 80 *vs.* 65 × 65 mm).

### 2.3 ^19^F MR of the polymer

#### 2.3.1 Relaxation properties

Fluorine *T*_1_ and *T*_2_ relaxation times of the polymer were measured using Minispec Mq60 relaxometer (1.5 T; Bruker Biospin, Germany). The polymer solution (*c*_pol_ = 70 mg mL^−1^) in 140 mM phosphate-buffered saline (PBS; Thermo-Fisher, Waltham, USA; pH = 7.4) was prepared in different concentrations in water and then placed inside the relaxation tube (*V* = 269 μL; Bruker, Germany) and heated in the water bath (*T* = 37.0 °C). *T*_1_ relaxation times were measured using the inversion recovery sequence with a biexponential fitting function,^32,33^ 4 scans, first/final pulse = 0.1/10 000 ms, 15 data points and 4 repetitions. *T*_2_ relaxation times were measured using the Carr–Purcell–Meiboom–Gill (CPMG) sequence with a biexponential fitting function, 4 scans, first/final pulse = 0.05/20 000 ms and 4 repetitions. The curves were fitted using minispec software. The tracer concentration (70 mg mL^−1^) and volume (269 μL) were similar to those used in further *in vivo* measurements.

#### 2.3.2 ^19^F MR spectroscopy and imaging

MRS and MRI of the tracer were performed using a custom-made dual ^1^H/^19^F coil on a 4.7 T MR scanner. The phantom used in the MR relaxation times measurement (2.3.1) was used to set the ^1^H and ^19^F Larmor frequencies (200.486 ± 1 kHz and 188.620 ± 1 kHz, respectively). First, ^1^H images were acquired for the phantom positioning and homogeneity examination using the following parameters: TR/TE = 3000/36 ms; turbo factor TF = 8; NA = 1; TA = 1 min 12 s; spatial resolution 0.18 × 0.18 × 6.0 mm^3^; FOV = 45 × 45 mm; digital matrix 256 × 256; number of slices 1 (transversal, sagittal and coronal plane). Subsequently, ^19^F MRS single-pulse sequence was used to optimize the fluorine frequency tuning and to acquire fluorine spectra (TR = 1069 ms; NA = 64; TA = 1 min 8 s). ^19^F MR images were acquired using the Rapid Acquisition with Relaxation Enhancement (RARE) sequence with the following parameters: TR/TE = 1000/43.5 ms; TF = 16; NA = 1 to 256; TA = 4 s to 17 min 4 s; spatial resolution 0.7 × 0.7 × 6.0 mm^3^; FOV = 45 × 45 mm; digital matrix 64 × 64; number of slices 1 (coronal plane). During the measurements, the temperature was set to 37.0 °C.

### 2.4 Experimental animals

All experimental protocols were approved by the Experimental Animals Welfare Committee of the Institute for Clinical and Experimental Medicine and the Ministry of Health of the Czech Republic (approval no. 36/2018) in accordance with the Protection of Animals against Cruelty Act (no. 359/2012) of the Czech Republic, which corresponds to the European Parliament and Council directive 210/63/EU. These experiments aimed at assessing the chemical stability, dissolution, and excretion of the implant under *in vivo* conditions and its biological compatibility for long-lasting applications. Lewis rats (LEW/Cr; attested by Envigo+, Huntingdon, United Kingdom) were provided by Animalab s.r.o. (Czech Republic) and fed with standard complete animal feed for laboratory animals (1324 mod. Velaz IRR, Czech Republic) purchased from Velaz, Ltd. (Czech Republic). The animals were kept in standard laboratory cages under a 12/12 light/dark regime in a conventional breeding facility with access to water and pelleted food *ad libitum*. During the injections and MR experiments, isoflurane (Baxter, Deerfield, United States) was used for anesthesia (5% isoflurane for induction, 1.0 to 1.5% for maintenance) and respiratory function was monitored with a trigger unit (Rapid Biomedical, Berlin, Germany).

Healthy male Lewis rats (*n* = 3) were administrated with the soluble polymer at the beginning of the experiment (henceforth referred to as day 0). The polymer was injected intramuscularly (IM) into the left hind leg and subcutaneously (SC) into the right hind leg (*V* = 200 μL, *c*_pol_ = 100 mg mL^−1^ in 140 mM PBS; pH was adjusted to 5.0 by adding concentrated hydrochloric acid). The combined thermo- and pH-responsive design serves to dissolve the polymer at pH 5.0, where the *T*_CP_ value is above the body temperature, to inject the polymer in the slightly acidic buffer into the body without needle obstruction or the need to use an organic solvent for injection. After implant administration, *in vivo* dissolution was studied by ^19^F MR with a dual surface coil used in previous *in vitro* studies.

In all MR measurements, the animals were placed in the coil holder with an anesthesia mask and eye cream (Ophthalmo-Septonex, Zentiva, Czech Republic) to avoid eye dryness during the procedure. Throughout the measurements, the rats were monitored for their welfare. In addition to weighing them, we collected blood samples using a catheter inserted into the tail vein from the onset of polymer administration^7^ and again 11 months after administration to confirm tracer biocompatibility. The blood samples were left to clot at room temperature for 20 minutes and then centrifuged (1610 g, 10 minutes); the resulting serum was removed, and biochemical blood markers were examined using a Fuji DRI-CHEM 500 multi-purpose, automatic, dry-chemistry analyser (Fujifilm, Tokyo, Japan) with the original commercial slides (LABtechnik s.r.o., Czech Republic). The concentration of bilirubin, creatinine, and albumin and the activity of alanine aminotransferase (ALT; EC 2.6.1.2) and aspartate aminotransferase (AST; EC 2.6.1.1) were assessed as standard blood markers.

#### 2.4.1 *In vivo* MR experiments

Following the shorter-term experiment described by Kolouchova, *et al.*,^7^ we performed a long-term *in vivo*^1^H/^19^F MR experiment. In this study, the rats were monitored using a more sensitive method (MRS), for 21 months and for 14 months using MRI. A phantom containing an aqueous solution of the polymer (*V* = 200 μL, *c*_pol_ = 100 mg mL^−1^; pH = 5.0) was placed between the hind legs to set the ^1^H and ^19^F frequencies at the given local field and to determine the 90° and 180° pulse frequency width. To determine the precise localization, ^1^H MRI was performed in three anatomical axes (sagittal, transverse and coronal) using a RARE sequence (TR/TE = 3000/36 ms; TF = 8; NA = 2; TA = 2 min 24 s; spatial resolution 0.25 × 0.25 × 1.5 mm^3^; FOV = 65 × 65 mm; digital matrix 256 × 256; number of slices = 15). To quantify the polymer signal, ^19^F MR spectra were measured (non-localized single-pulse sequence; TR = 1000 ms; NA = 32/64; TA = 1 min 8 s) with and without a phantom reference and without relocating the rats. If the ^19^F MRS signal had sufficient SNR, ^19^F MRI was performed in the coronal axis using a RARE sequence (TR/TE = 1000/43.5 ms; TF = 16; NA = 512; TA = 17 min 4 s; spatial resolution 1.0 × 1.0 × 13 mm^3^; FOV = 65 × 65 mm; digital matrix 64 × 64; number of slices = 3) to assess more details on the implant location, volume and signal intensity.

#### 2.4.2 Histological examination

At the end of the experiment, rats were sacrificed by anesthetic overdose, and the tissue from the injection sites was visually inspected for possible pathological alterations. Their liver, spleen, kidneys, injected muscle, and contralateral muscle were sampled for histological examination. These tissues were fixed in 10% buffered formaldehyde for 2 days and then embedded in paraffin, after which 5 mm-thick sections were stained according to standard haematoxylin & eosin and Verhoeff-van Gieson stain procedures.^34^ All samples were compared with healthy rat histology.

### 2.5 MR data evaluation

Implant dissolution was quantified using ^19^F MR data. We performed a volume and signal intensity analysis based on manual segmentation from images of the implants and calculated the signal- (SNR) and contrast-to-noise ratio (CNR) and the integral changes of the spectroscopic signals. All MR results are presented as percentage values of day 0, that is, the first MR measurement after polymer injection. For quantification, the images were post-processed with the same weight using a sine-squared filter implemented in ParaVision 4.0 software (Bruker, Germany) on a 4.7 T scanner console.

The imaging properties of the implant were assessed by ^19^F MRI using ImageJ 1.48 (National Institutes of Health, Bethesda, USA). Its volume was calculated as milliliters, according to the measurement parameters, as slice thickness, FOV and image matrix. To variation of both ^19^F MRI signal intensity and volume as a function of time was expressed as dynamic changes (DCH) because this parameter minimizes deviation in a small group and represents the actual state of the implant during dissolution by multiplying SNR by volume:

1 DCH = *V* × SNR

^19^F MRI SNR was calculated as2
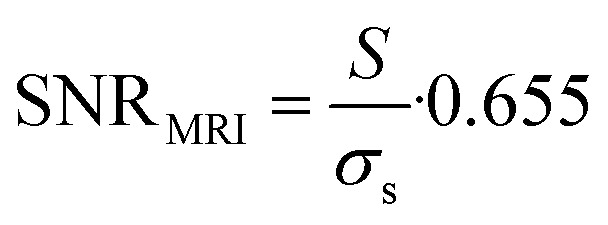
where *S* is signal intensity in the region of interest (ROI), *σ* is the standard deviation of background noise, and the constant 0.655 reflects the Rician distribution of background noise in a magnitude MR image.^35^ In addition, CNR was calculated as a difference between fluorine SNR (SNR_1_) and surrounding SNR (SNR_2_):

3 CNR = SNR_1_ − SNR_2_

where both SNRs were measured as shown in eqn (2).

MR spectra were processed using a Matlab script; within post-processing, Lorentz-Gauss apodization was used for noise suppression in the spectra, and Fast Fourier Transform (FFT) was used to transform the MR signal into the frequency domain. Data were assessed in the following steps: (1) the noise region of the spectrum was selected, and the mean noise value was calculated, (2) the signal peak area was selected (the region where the signal was at least twice higher than noise value), (3) the point at which the signal reached the mean noise value was marked as the end of the evaluated signal area.

The noise was analysed in the interval between the end of the acquired fluorine signal of the polymer and the edge of the fluorine spectrum using the same frequency width for both noise and signal regions. SNR was calculated from the resulting absolute values of spectra integral divided by the noise and multiplied by the Rician distribution of the background noise:4
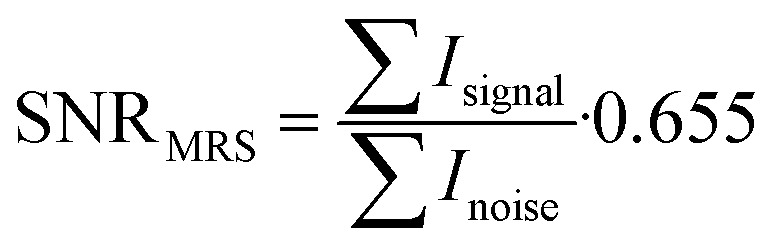


In addition to SNR, the ^19^F MRS signal change over time was expressed as the percentage of the ^19^F signal integral area minus the noise integral.

^19^F MRS and ^19^F MRI data were fitted to a monoexponential function using OriginPro 2018 (b9.5.1.195, OriginLab Corporation, Northampton, MA, USA):

5 *I* = *I*_0_e^−*kt*^

where *I*_0_ (initial signal) and *k* (dissolution kinetics constant) are parameters, *t* (time) is an independent variable and *I* (overall signal) is the dependent variable. Subsequently, we calculated the biological half-lives (*T*_1/2_) of the polymer signal (based on ^19^F MRS data) and the depot volume dissolution (based on ^19^F MRI data) using the following equation:6
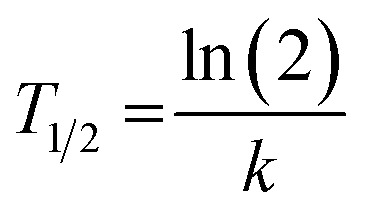


Lastly, we calculated the overall polymer biological half-lives from the individual fits (individual animals) as pooled variance weighted by the number of fitted points in each animal.

## 3. Results and discussion

### 3.1 Injectable polymeric tracer

**Scheme 1:**
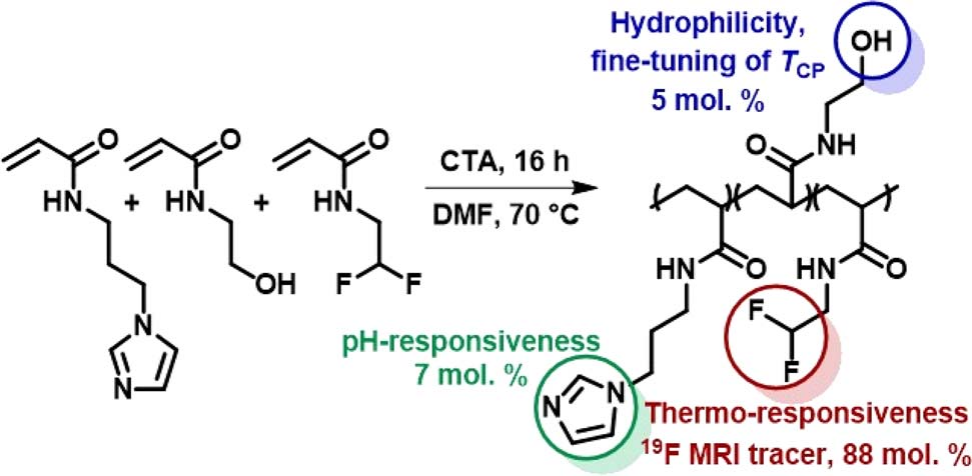
Synthesis of the thermoresponsive copolymer P(DFEAM-*co*-ImPAM-*co*-HEAM), which forms a solid implant *in vivo* upon injection followed by temperature and pH increase.

The thermoresponsive polymeric tracer was synthesized *via* reversible addition–fragmentation chain transfer (RAFT) copolymerization of the corresponding monomers as previously described in Kolouchova, at al (Scheme 1).^7^ The final copolymer consisted of PDFEA units (88 mol%), *N*-[3-(1*H*-imidazole-1-yl)propyl]acrylamide (ImPAM, 7 mol%), and *N*-(2-hydroxyethyl)acrylamide (HEAM, 5 mol%). The DFEA monomer contained fluorine atoms for ^19^F MR tracing and endowed our polymer with thermoresponsive properties; the imidazole moieties of ImPAM introduced pH-responsiveness, whereas the hydrophilic monomer HEAM was used to modulate the LCST of the polymer. The copolymer showed thermoresponsive behavior in aqueous solutions. Despite being completely soluble at lower temperatures, the polymer showed macroscopic phase separation when heating its solution above its *T*_CP_, forming a solid implant.

To facilitate implant administration, the *T*_CP_ value was strongly pH-dependent. The variation of *T*_CP_ with pH was described in Kolouchova, *et al.*^7^ The polymer solution was administered at pH = 5, where the *T*_CP_ was above body temperature (60 °C). Conversely, at physiological pH = 7.4, the *T*_CP_ dropped below body temperature to 27 °C, thus ensuring efficient solid implant formation. The molar mass of the polymer (*M*_w_ = 37.8 kg mol^−1^) and its low dispersity (*Đ* = 1.12) should enable its renal elimination upon gradual implant dissolution.^36^

### 3.2 ^1^H/^19^F MR coil homogeneity

**Fig. 1:**
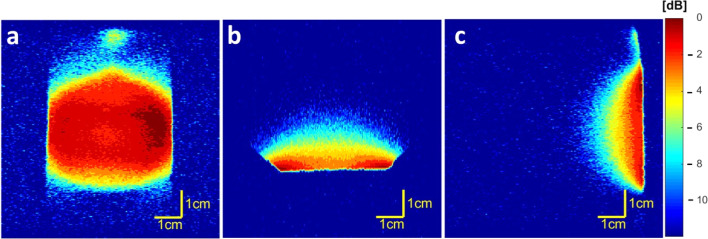
Coil homogeneity measured using ^1^H MRI on 4.7 T scanner in (a) coronal (b) axial and (c) sagittal planes; the color scale reflects signal attenuation (dB) – from the lowest (red) to the highest (blue).

Coil homogeneity testing revealed that the magnetic field is homogeneous in the FOV intended for further *in vivo* measurements of injection sites in an adult rat (Fig. 1). In the coronal plane (Fig. 1a), the signal decreased by 4 dB on the border area exceeding the FOV range used for *in vivo* imaging. Furthermore, low attenuation (0 to −3 dB) was observed in the required height (Fig. 1b) and depth (Fig. 1c) from the coil surface.

### 3.3 ^19^F MR of the polymer

#### 3.3.1 Relaxation properties

Measurements of the *T*_1_ and *T*_2_^19^F relaxation times of the polymer, in its aggregated form (*T* = 37.0 °C, pH = 7.4), were processed by monoexponential and biexponential fitting (Table 1). Differences in *T*_1_ and *T*_2_ values for monoexponential curve fitting and biexponential fitting matched the phase changing of the polymer during the measurement. Biexponential fitting provided us with additional information on the MR properties of the polymer, because the temperature and thus the physical state of the tracer may slightly change during data acquisition. The favorable ^19^F relaxation values allowed us to continue using MR methods with high sensitivity.

**Table 1:** ^19^F Relaxation times of the polymer in aggregated form (*T* = 37.0 °C, pH = 7.4) with mono- and biexponential curve fitting measured at 1.5 T relaxometer

^19^F relaxation time	Curve fitting	*T* ± SD [ms]
*T*_1_	Monoexponential	221.5 ± 2.5
	Biexponential	186.5 ± 6.2/925.0 ± 150.0
*T*_2_	Monoexponential	80.3 ± 3.3
	Biexponential	6.1 ± 0.2/170.5 ± 1.7

#### 3.3.2 ^19^F MR spectroscopy and imaging

**Fig. 2:**
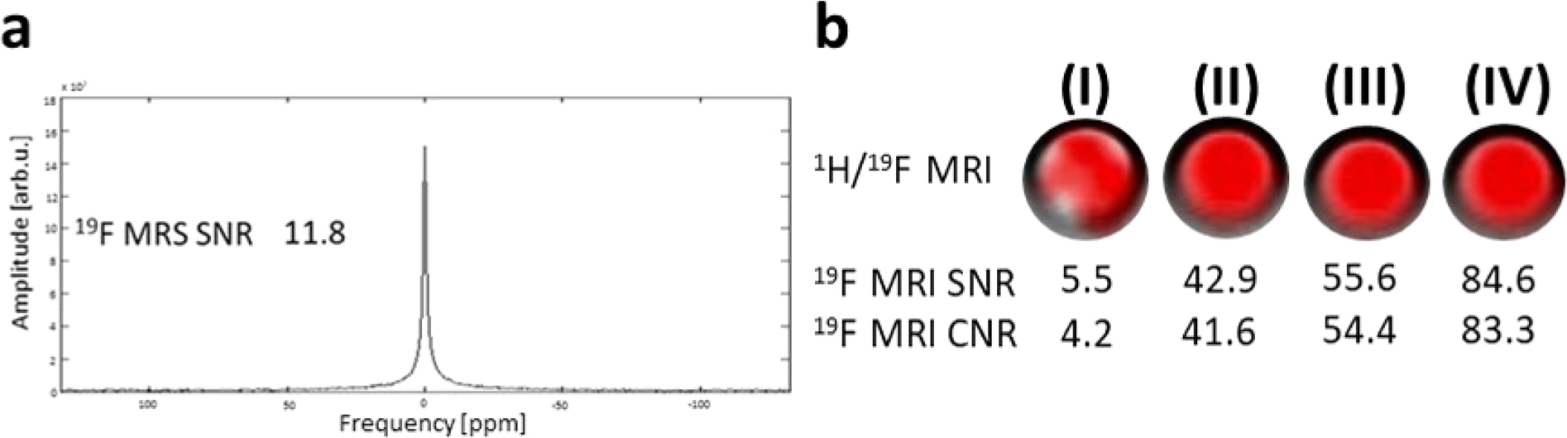
Results of phantoms' (*c*_pol_ = 70 mg mL^−1^) ^19^F MR signal measured on 4.7 T scanner. (a) ^19^F MR spectrum of the polymer measured with 64 acquisitions (TA = 1 min 8 s) with given SNR and with fluorine signal peak at 0 ppm; (b) overlaid ^1^H/^19^F MRI (red – ^19^F signal) with given ^19^F SNR and CNR for TA = 4 s (I) – 17 min 4 s (IV).

^19^F MRS measurements of the polymer solution demonstrated that the fluorine signal can be detected in a very short measurement (time of acquisition TA = 2 s). However, to calculate a more relevant SNR for subsequent *in vivo* measurements, we used a longer acquisition time (Fig. 2a; TA = 1 min 8 s; SNR = 11.8). ^19^F MRI results (Fig. 2b; TA = 4 s – 17 min 4 s) provided high SNR (5.5 to 84.6) and CNR (4.2 to 83.3) values, which increased with the measurement time.

### 3.4 *In vivo* ^19^F MRS/MRI

**Fig. 3:**
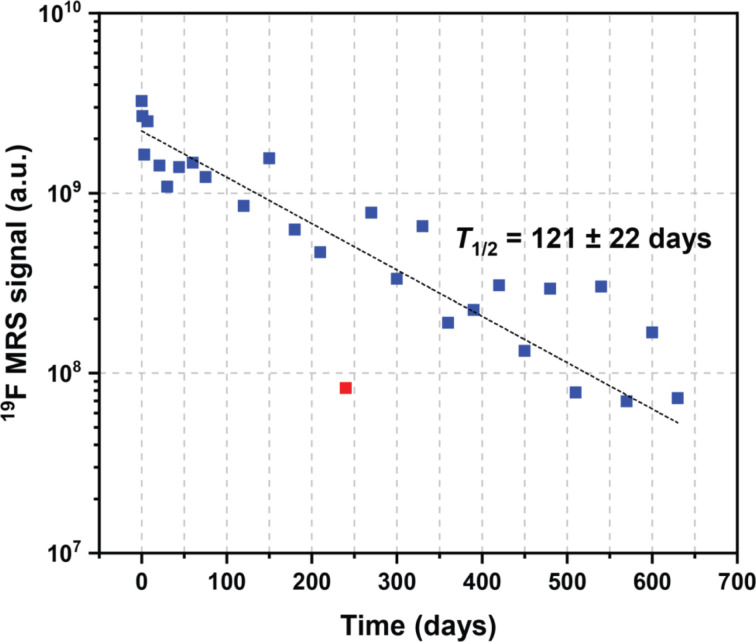
Example of expected values of the ^19^F MRS signal biological half-life extrapolated from kinetics trends.

Both fluorine spectroscopic signal intensities (^19^F MRS) and depot volumes (^19^F MRI) showed first-order kinetics of dissolution with ^19^F MRS biological half-lives of approximately 123 ± 24 days (Fig. 3, S1 and S2†). We used ^19^F MRS data to describe the long-term behaviour of the implant (Table S1†), because imaging is more prone to be affected by the geometry of the implants.^7^ Our results indicate that the polymer remains at the administration site for a long and predictable time and is suitable for long-term purposes.

**Fig. 4:**
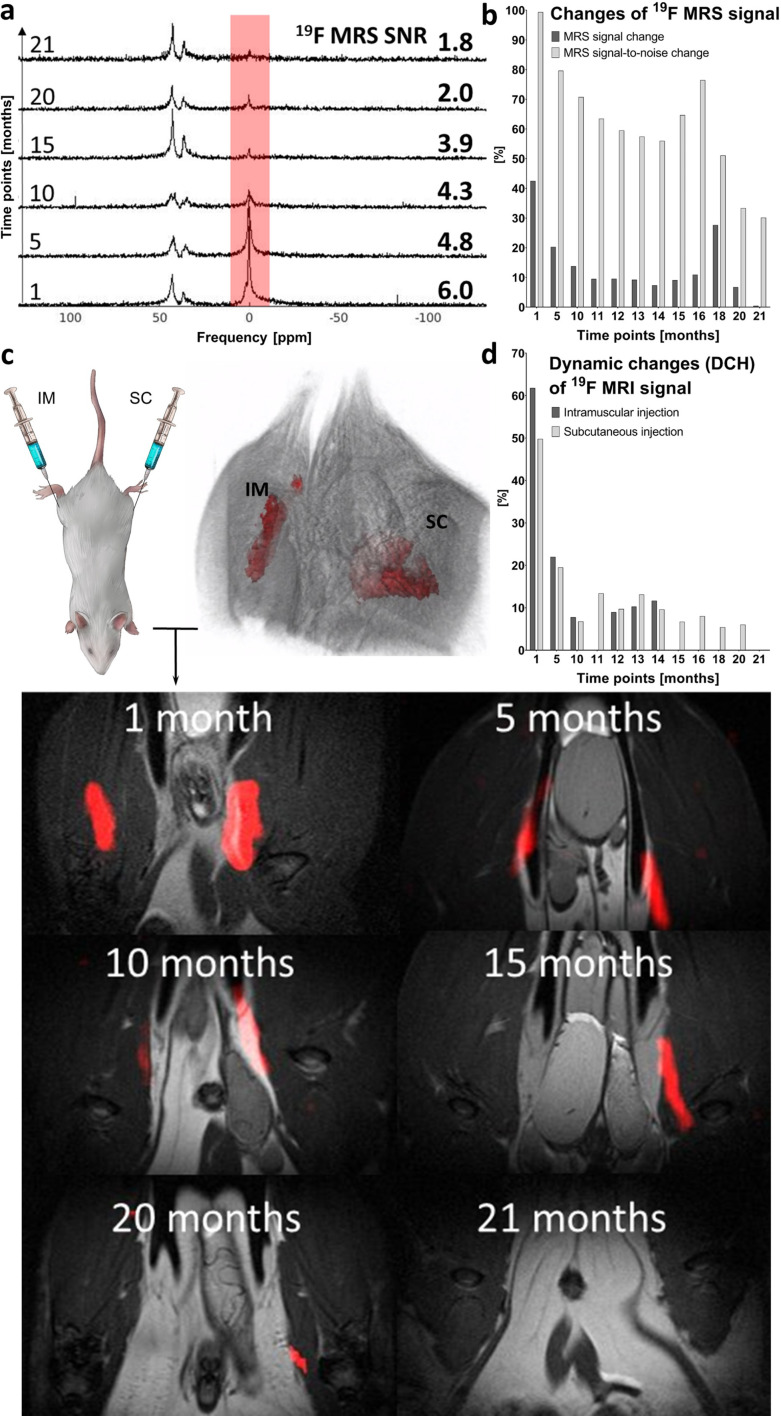
Long-term *in vivo*^19^F MR measurement on a 4.7 T scanner. (a) *In vivo*^19^F MR spectrum in time with varying SNR values; polymer signal peak at 0 ppm and isoflurane signals at 36 and 43 ppm. (b) ^19^F MRS percentage changes of signal with subtracted noise and SNR. (c) Injection sites with 3D MRI reconstruction; overlaid ^1^H (grayscale) and ^19^F (red) MRI of IM and SC depots in the coronal plane at various time points and (d) DCH of ^19^F MRI signal from injection sites. All results in (b) and (d) are expressed as a percentage of the day 0 signal (100%) and represent the signal change over time (from 1 to 21 months after administration).

The polymer signal remained detectable for up to 14 months using ^19^F MRI and for up to 21 months using ^19^F MRS. ^19^F MRS SNR ranged from 99 to 30% (signal intensity at day 0) from 1 to 21 months post-injection. Once the noise was subtracted, the signal ranged from 43 to 0.4% in the same period. Spectroscopy results are shown in Fig. 4a and b, and detailed data on SNR are outlined in Table S2.† Although isoflurane anesthetic contains fluorine atoms, their ^19^F MR chemical shift is significantly different from that of the implant (Δ*δ* = 36 and 43 ppm). Therefore, the isoflurane anesthetic does not interfere with the signal of the implants *in vivo*.

Long-term *in vivo*^19^F MRI revealed that the signal detection period varies with the injection site. The signal was detected for 14 months after IM implant administration and for 20 months after SC injection (Fig. 4c and d). All results are expressed as percentage of signal intensity at day 0 and represent the change in time. Statistical analysis revealed a significant difference (*p* < 0.01) between all paired (1–14 months) ^19^F MRI SNR percentage changes from both injection sites.

Implant performance was assessed based on DCH (Fig. 4d). This MRI-based parameter reflects changes in volume and SNR on both IM and SC injection sites. Despite minor signal fluctuations, DCH shows a clearly decreasing trend (in line with the kinetics of dissolution). At full dissolution time, DCH reached 12.0% of its initial value on the IM injection site (14 months) and 6.0% on the SC site, with a longer dissolution time (20 months). Implant volume and SNR changes assessed by ^19^F MRI are summarized in Table S3.†

### 3.5 Biocompatibility

Prior to the *in vivo* experiments, the cytotoxicity of the polymer was tested on various cell lines. No toxic effect was found in the range of polymer concentrations (8 to 1000 μg mL^−1^) used in a previous study.^7^ Those results were confirmed in *in vivo* experiments; the animals' weight and standard plasma biomarkers remained at normal levels.^7^ Over time, the animals gained weight, which increased from 500 (10 months after injection; rats at the age of 15 months) to 651 (21 months after injection; rats at the age of 26 months) grams on average.

**Fig. 5:**
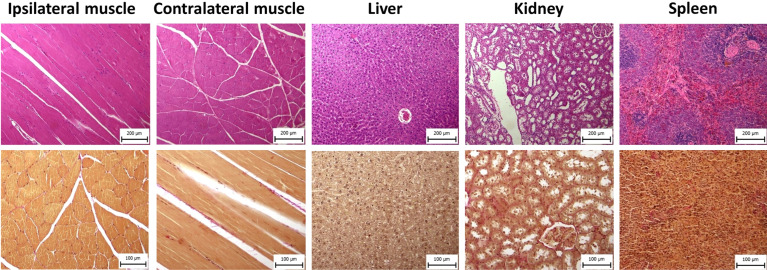
Histological images of injected muscle, control muscle, liver, kidney, spleen; tissue was collected 12 months after polymer administration and stained using haematoxylin & eosin (upper row; magnified 100-fold) and Verhoeff-van Gieson stain (lower row; magnified 200-fold).

We assessed plasma biomarkers of liver and kidney damage and performed a histological examination to ascertain potential long-term polymer effects on animal welfare. All biomarker values were within the ranges measured in the control group (Fig. S3†). Histological examination of the injection sites and selected organs did not reveal any pathological changes in the tissue comparing with the normal tissue (Fig. 5). In skeletal muscle, parallel muscle fibers showed normal size and preserved architecture, with no atrophy, interstitial fibrosis and/or inflammatory reaction. Liver tissue had preserved lobular architecture without fibrotic expansion of portal triads, metabolic changes (steatosis), cholestasis or necroinflammatory activity. The renal cortex and medulla showed a preserved architecture; glomeruli were normal in size, with no significant interstitial fibrosis or inflammation. The spleen showed mild white pulp activation, secondary hemosiderosis, and foci extramedullary hematopoiesis in the red pulp. No significant fibrosis was identified by Verhoeff-van Gieson (V-v G) staining.

### 3.6 Discussion

The implant-forming injectable polymeric system used in this long-term *in vivo* study was based on thermo- and pH-responsive copolymers. The *in vivo* dissolution and excretion of the implants were studied using ^19^F MR techniques. Following our previous study,^7^ we studied the long-term safety and dissolution pharmacokinetics of the most prominent thermoresponsive polymer and discuss the behavior of the implants under *in vivo* conditions for 21 months.

The reproducibility and quality of the measurements may be attributed to the MR scanner hardware. Radiofrequency coils are an essential MR hardware component as they directly affect spatial and temporal resolution, sensitivity and uniformity in MRI.^37^ In this study, a custom-made coil was tested for its sensitivity – establishing the homogeneity (Fig. 1) with very low signal attenuation (−3 dB) in the coronal plane, which was used for *in vivo* imaging quantification. The coil area (diameter of 4 cm) covers both injection sites in adult rats, and the location of the implant site favors good magnetic field homogeneity, which provides high SNR and accurate measurements. A dual resonator coil could be used to further improve the homogeneity,^14^ but doing so would worsen the sensitivity, which is crucial for fluorine detection.

We also determined the parameters of the tracer by measuring the *in vitro* MR relaxation times (1.5 T) and by MR imaging and spectroscopy (4.7 T) of the phantoms. Both experiments were performed in aggregated polymer state (*T* = 37 °C; PBS buffer, pH 7.4) and at magnetic fields close to those used in clinical practice, thus providing relevant information for its future medical applications. Phantom ^19^F MR imaging and non-localized spectroscopy confirmed that the polymer displays good sensitivity with a high SNR at short acquisition times. MR properties of the polymer such as favorable relaxation times and high sensitivity were essential for further long-term *in vivo* signal detection.

We evaluated implant dissolution *in vivo* by both ^19^F MRS and ^19^F MRI, monitoring the total signal of the polymer and its volume, respectively. To compare the results at different time points, we expressed the data as the percentage of MR signal intensity after tracer administration (day 0). As a more sensitive method (detectable long after MRI signal is lost), non-localized spectroscopy reflects subtle changes in the dissolution on both injection sites, thereby providing us with precise and hence more predictable information (see kinetics).

^19^F MRS analysis revealed a slow decrease of the implant signal over time. SNR from the spectra exceeded its baseline value on the first day after fluorinated polymer administration. This effect could be associated with the accuracy of the method due to tracer accumulation at a small volume with a high concentration, while forming an implant,^38^ or with polymer binding to the extracellular matrix, as already mentioned.^7^ This property is presumably caused by the low content of hydrophilic units in the polymer structure, responsible for tuneable implant dissolution.

^19^F MR spectra and imaging may be affected by differences in *in vivo* polymer binding over time under changing conditions (temperature and pH). This effect may explain minor deviations in signal detection, which was recovered after being undetectable in the previous measurement. This effect may also be reflected in the *T*_1_ and *T*_2_ MR relaxation time curves with biexponential fast and slow relaxations, strengthening the hypothesis that the short relaxation component of this tracer derives from polymer–tissue interactions. By binding to the extracellular matrix, the tracer maintains a sharp signal, which may point out faster relaxation of some molecules. We were able to visualize only the fluorine component with slower relaxations due to the longer echo time (TE = 43.5 ms) used for the imaging sequence. These components represent the polymer state (changing from liquid to solid-implant form) and changes within its *in vivo* bonding to the extracellular matrix dictated by the copolymers, surrounding temperature and tissue binding.

As shown in Fig. 4, the decrease in the ^19^F MR signal of the implant attributed to polymer dissolution has only a negligible effect on chemical shift and peak shape. Additionally, the implant resonance frequency was significantly shifted from isoflurane (Δ*δ* = 36 and 43 ppm, Fig. 4). Therefore, the isoflurane signal does not interfere with the signal of the implant in ^19^F MR, which is crucial for its *in vivo* use.

Both polymer signal (^19^F MRS) and implant depot volumes (^19^F MRI) decreased mono-exponentially over time. The biological half-lives of the polymer were similar in all rats (Fig. S2†), with the overall biological half-life reaching 124 ± 25 days (mean ± pooled variance of all polymers), based on spectroscopic data. Furthermore, we used these dissolution kinetics data to calculate the expected signal at various time points, as outlined in Table S1.† Lastly, implant volumes (determined from ^19^F MRI data) continuously decreased throughout the experiment, but the data were too scattered for a rigorous pharmacokinetics analysis. Thus, the results indicate that the polymer remains at the administration site for a long time.

We quantified DCH (*V* × SNR) to assess implant dissolution based on volumetrics because this parameter covers two key factors: implant volume and intensity. Thus, DCH provides data on noise-related implant dynamics. This approach ensures a precise signal analysis and identifies subtle changes in the signal, as well as background noise. DCH also enabled us to perform a more robust comparison between injection sites with higher correlation (*r* = 0.80) between them than MR signal (*r* = 0.69) or volume (*r* = 0.64) alone. SNR assessed by MRI and MRS over time showed *r* = 0.64 and *r* = 0.61 for IM and SC injection, respectively.

*In vivo* ^19^F MRI quantification from the raw data revealed that the implants dissolved more quickly upon IM than upon SC administration possibly due to the IM injection site. The implant was formed in the inner side of the thigh, where higher vascularization favors the absorption rate and the depot spread over a wider tissue area. IM administration is preferred in many animal models because the muscle tissue has a greater blood supply, thereby accelerating absorption;^39^ nevertheless, this polymer can be injected locally to any body site, forming an implant under physiological conditions.

Our multiresponsive injectable polymer can be characterized as a stable *in vivo* implant with excellent MR properties and very slow dissolution under *in vivo* conditions the molar mass of the polymer implant is lower than the renal filtration threshold (approximately 40 kDa). Based on this property and on the positive charge of the polymer, the polymer should be eliminated through urine after implant dissolution.^7,40^ This assumption will be researched in detail in further excretion studies with the analysis of urine and/or feces, together with the possible accumulation in other organs. The biocompatibility results confirm the negligible long-term influence of the tracer and the general welfare of the animals subjected to a long exposure.

Thermoresponsive materials are capable of self-assembly and disassembly as a function of solution temperature past their *T*_CP_ and thus are extensively studied for various medical applications.^41^ pH sensitivity could be used to release the co-administrated drug because the pH is lower in pathological tissues, such as solid tumors, or upon inflammation.^42,43^ Long-term analysis of *in vivo* implant dynamic changes may enable future clinical tests of the polymer with a slow release of co-administrated drugs in the physiological environment.^13^ The long-term stability of the this release may be a significant advantage over drugs administered in acute oral form or by injection as their blood levels may rise above and fall below optimal therapeutic values with each dose.^25^

Considering the problems discussed above, many studies have focused on local controlled release.^6,7,15,16,20–29^ In this context, non-invasive imaging and theranostics is an emerging area of research. These implants may also be administered to patients requiring conventional chemotherapy with a low targeting efficacy and side effects,^29,30^ thereby improving the quality of care^31^ while reducing local inflammation and avoiding stroke.^22^

## 4. Conclusions

Our ^19^F MR monitoring of slowly dissolving fluorinated stimuli-responsive polymeric implants formed upon injection into healthy rats highlights their favorable MR relaxation times, high specificity and sensitivity and long dissolution, all excellent properties for *in vivo* long-term applications. This tracer shows potential as a next-generation injectable implant because no adverse effect was assessed after a long and intensive exposure to this implant, with all major biochemistry indices remaining at levels similar to those of the control. Moreover, histological examination shows no pathological alterations in tissues upon tracer administrations. These findings open up a new path towards medical applications of ^19^F MR image-guided biomaterials requiring long-term administration at a specific site.

## Author contributions

Natalia Ziółkowska: formal analysis, investigation, writing – original draft, visualization, methodology. Martin Vít: investigation, software. Ondřej Groborz: formal analysis, writing – review & editing, visualization. Kristýna Kolouchová: resources, writing − review & editing, methodology. David Červený: formal analysis, software. Ondřej Sedláček: conceptualization, methodology, writing – review & editing. Daniel Jirák: conceptualization, methodology, writing – original draft, supervision, resources, project administration, funding acquisition.

## Conflicts of interest

The authors declare that they have no known competing financial interests or personal relationships that could have appeared to influence the work reported in this paper.

## Supplementary Material

NA-006-D4NA00212A-s001
