# Supplementary material for: Long-term in vivo dissolution of thermo- and pH-responsive, 19F magnetic resonance-traceable and injectable polymer implants
Source: Nanoscale Adv. 2024 Apr 8;6(12):3041–51. doi: 10.1039/d4na00212a (PMC11166117; doi:10.1039/d4na00212a)
Supplement: NA-006-D4NA00212A-s001 [file NA-006-D4NA00212A-s001.pdf]

Electronic Supplementary Material (ESI) for Nanoscale Advances.
This journal is © The Royal Society of Chemistry 2024

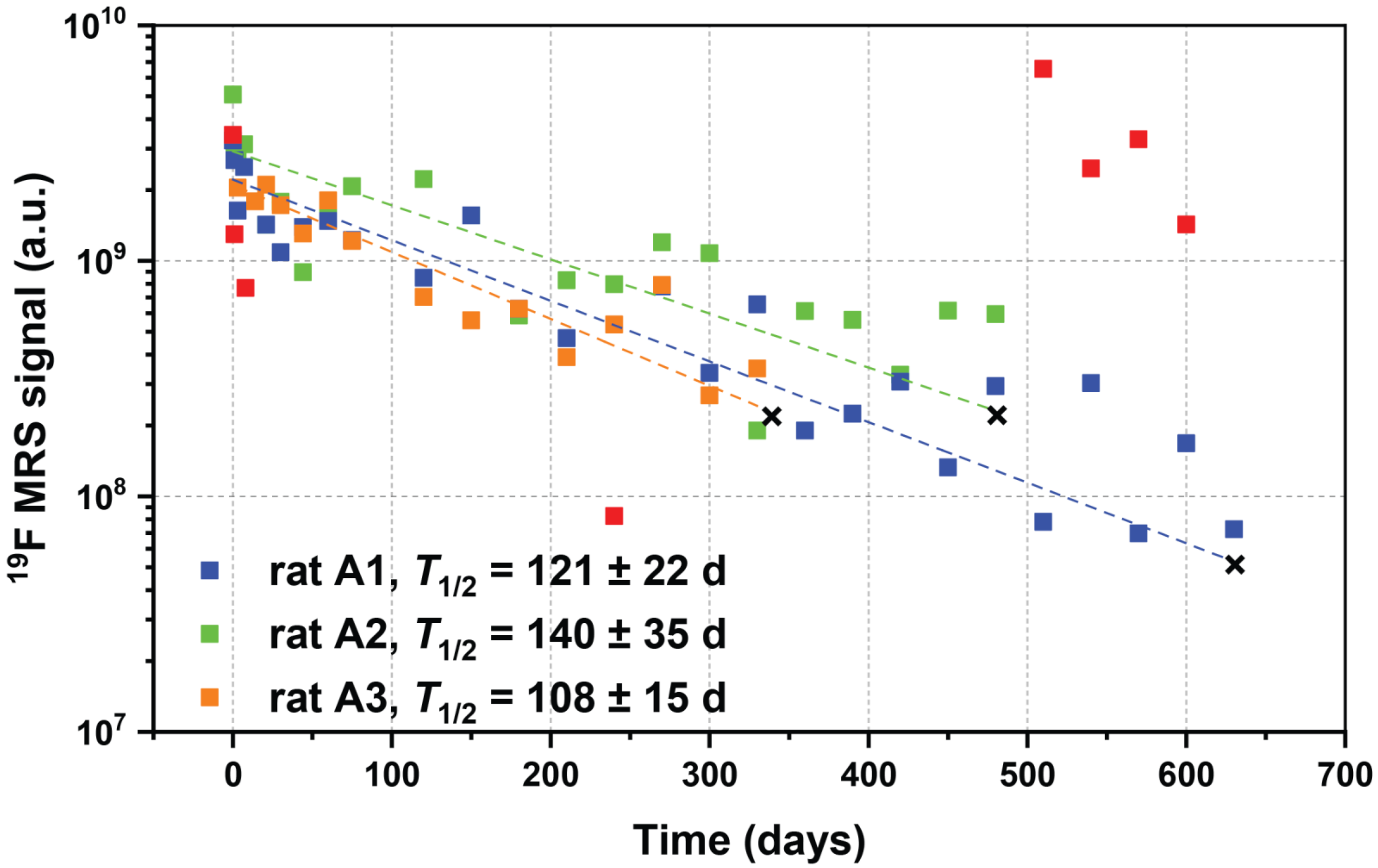

Fig. S1. ^{19}F MRS signal of a depot in all rats presented as a function of time fitted with a monoexponential curve; outlier points are marked in red.

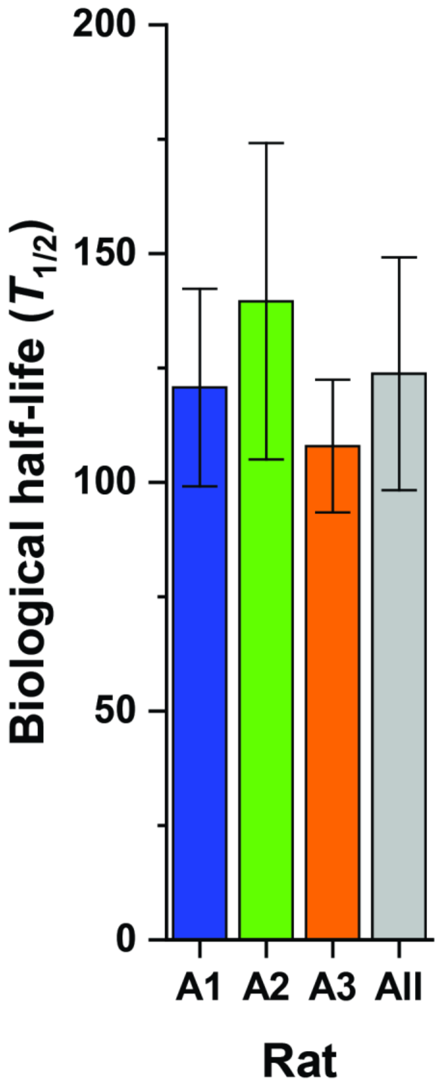

Fig. S2. Biological half-lives of the polymer in all rats based on ^{19}F MR spectroscopic data (mean ± pooled variance).

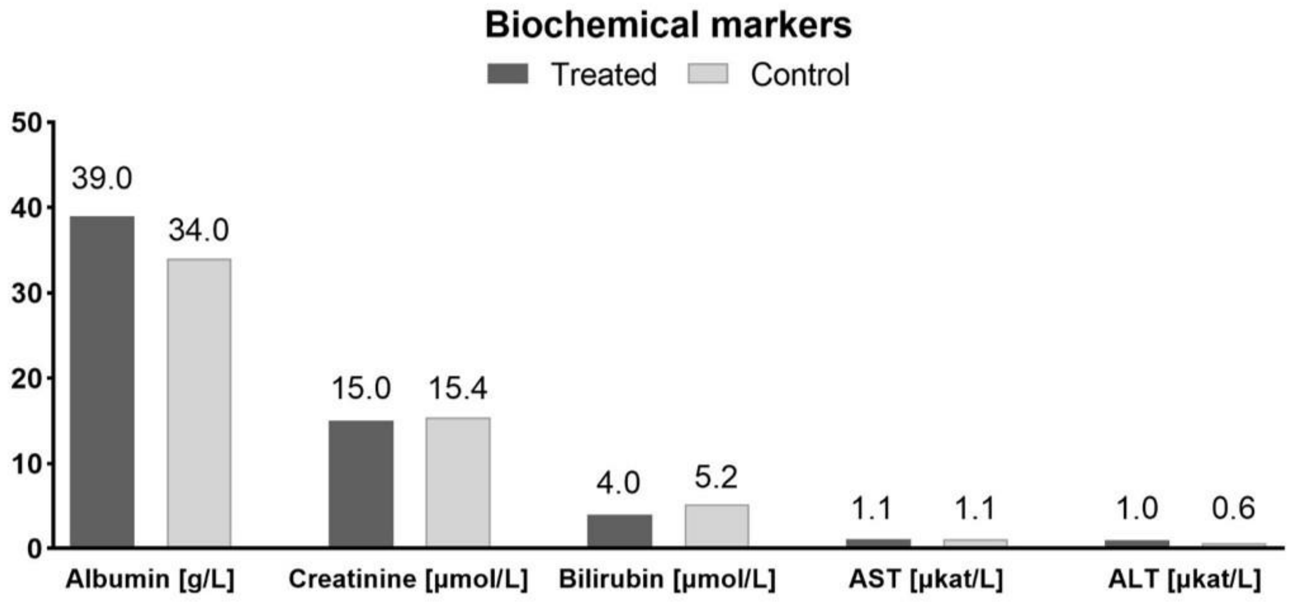

Fig. S3. Results of tested blood biomarkers of systemic toxicity. Activity of alanine aminotransferase (ALT) and aspartate aminotransferase (AST), total concentration of bilirubin, creatinine and albumin were determined from the blood serum. Results from the treated and control group are presented.

Table S1. Expected values of ^{19}F MRS signal extrapolated from kinetics trends, expressed as a percentage of initial signal (day 0).

**Time**	**Signal (%)**
14 days	92 ± 1
1 month	84 ± 3
3 months	59 ± 6
5 months	42 ± 7
10 months	18 ± 6
15 months	8 ± 4
20 months	4 ± 2
30 months	1 ± 1

Table S2. The results of ^{19}F MR non-localized spectroscopy (signal, noise, signal-noise and signal-to-noise SNR). The mean fluorine signal integral of the ^{19}F MR spectra in different time points following the injection (1 day – 21 months) detected on 4.7 T scanner and calculated using Matlab script. The results are given in percentage of the day 0 (100 %). D – day, M – month.

Time point	1 D	1 M	5 M	10 M	11 M	12 M	13 M	14 M	15 M	16 M	17 M	18 M	19 M	20 M	21 M
Signal [%]	64.3	42.7	20.8	14.3	10.1	10.2	10.0	8.1	9.5	11.3	84.4	35.3	42.8	20.4	1.9
Noise [%]	61.1	43.8	25.1	18.9	15.5	16.1	16.8	14.6	13.2	14.6	75.5	99.6	91.6	74.5	6.2
Signal–noise [%]	64.6	42.5	20.3	13.8	9.5	6.4	6.1	4.9	6.1	7.3	52.4	18.4	23.2	4.5	0.4
SNR [%]	104.8	99.4	79.6	70.8	63.5	59.5	57.5	56.0	64.7	76.5	43.9	51.1	30.6	33.4	30.1

Table S3. The results of ^{19}F MR imaging (relative volume of the ^{19}F signal, singal-to-noise ratio SNR, contrast-to-noise ratio CNR and dynamic change DCH: VxSNR). Results were obtained from intramuscularly (IM) and subcutaneous (SC) injections on 4.7 T scanner from different time points following the injection (1 day – 21 months). The results are given in percentage of the day 0 (100 %). D – day, M – month; N/A – not applicable.

Time point	1 D		1 M		5 M		10 M		11 M		12 M		13 M		14 M		15 M		16 M		17 M		18 M		19 M		20 M		21 M	
Injection site	IM	SC	IM	SC	IM	SC	IM	SC	IM	SC	IM	SC	IM	SC	IM	SC	IM	SC	IM	SC	IM	SC	M	S	M	S	M	S	M	S
Volume [%]	34.9	25.8	100.6	35.7	36.0	13.2	20.3	12.5	0	18.8	17.4	19.2	19.2	20.8	21.8	13.8	0	8.2	0	14.4	0	8.2	0	11.8	0	8.7	0	12.8	0	0
SNR [%]	55.8	58.1	51.4	146.9	47.7	93.3	16.1	51.1	N/A	75.4	32.4	53.4	33.8	62.3	33.8	70.9	N/A	40.8	N/A	52.7	N/A	55.1	N/A	46.6	N/A	27.3	N/A	45.8	N/A	N/A
DCH [%]	14.8	14.7	61.8	49.8	22.0	19.5	7.8	6.8	N/A	13.4	9.0	9.7	10.3	13.1	11.7	9.6	N/A	6.7	N/A	8.0	N/A	4.5	N/A	5.4	N/A	4.8	N/A	6.0	N/A	N/A
CNR [%]	43.0	51.7	44.5	155.0	40.7	98.6	11.3	42.9	N/A	71.2	26.2	45.9	28.0	56.5	28.6	66.5	N/A	39.9	N/A	45.1	N/A	45.6	N/A	37.6	N/A	23.2	N/A	36.9	N/A	N/A
